# Supplementary material for: Variation in morpho-physiological and metabolic responses to low nitrogen stress across the sorghum association panel
Source: BMC Plant Biol. 2022 Sep 9;22:433. doi: 10.1186/s12870-022-03823-2 (PMC9461132; doi:10.1186/s12870-022-03823-2)
Supplement: Supplementary file 1 — Table S3. and Figures S1-10. [file 12870_2022_3823_MOESM1_ESM.pdf]

**Table S3.** **Summary of the PLSR results**

| Trait | $n_{LV}$ | Training $R^2$ | Training RMSE | Validation $R^2$ | Validation RMSE |
|---|---|---|---|---|---|
| CHL | 19 | 0.82 | 48.73 | 0.83 | 46.22 |
| SLA | 10 | 0.62 | 6.47 | 0.76 | 5.97 |
| N | 16 | 0.66 | 0.29 | 0.46 | 0.34 |
| P | 10 | 0.18 | 0.06 | 0.25 | 0.07 |
| K | 10 | 0.34 | 0.34 | 0.22 | 0.37 |

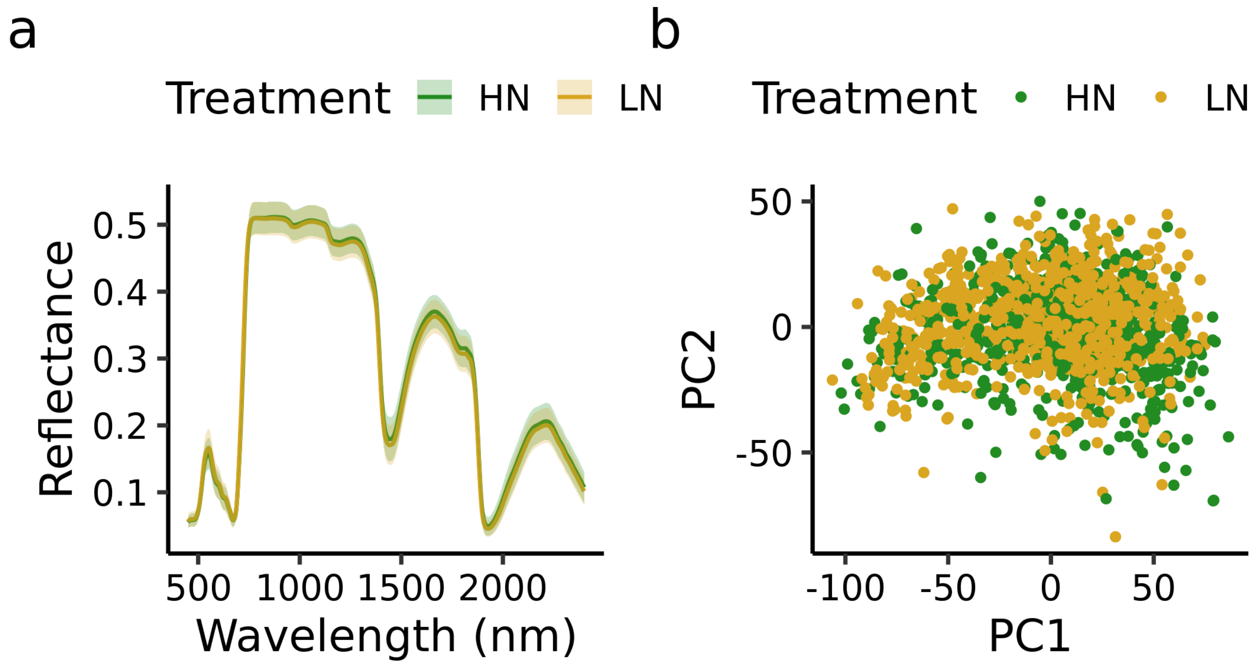


**Figure S1.** **a** The mean leaf hyperspectral of the sorghum plants from high nitrogen(HN; green) and low nitrogen(LN; yellow). The bounding envelopes are the standard deviation. **b** Principal component score for individual plat (PC1 vs. PC2).

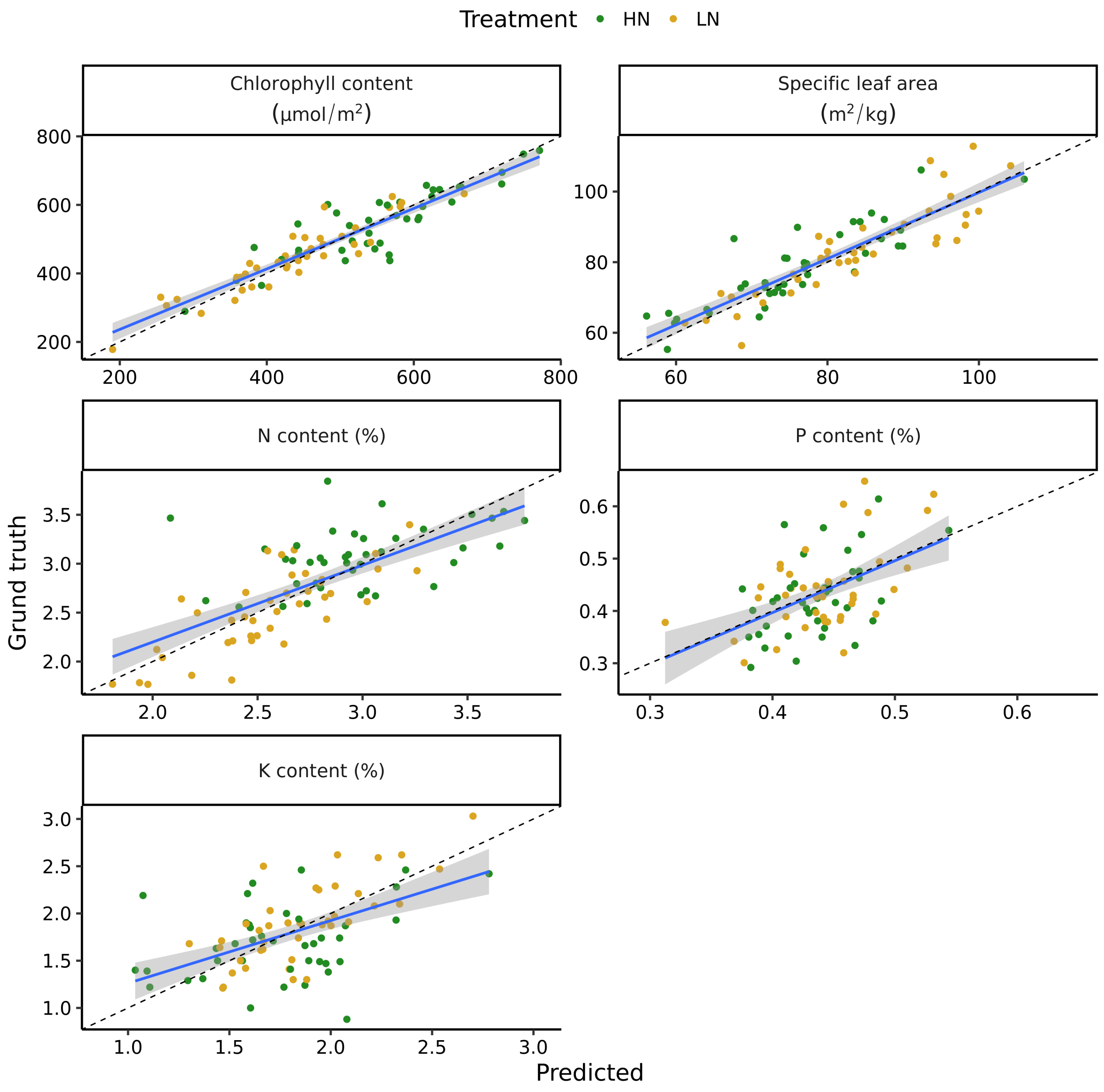


**Figure S2.** Scatter plots of ground truth and predicted values for training set sorghum leaves. Statistics for prediction can be found in Table S3. HN - high nitrogen, LN - low nitrogen.

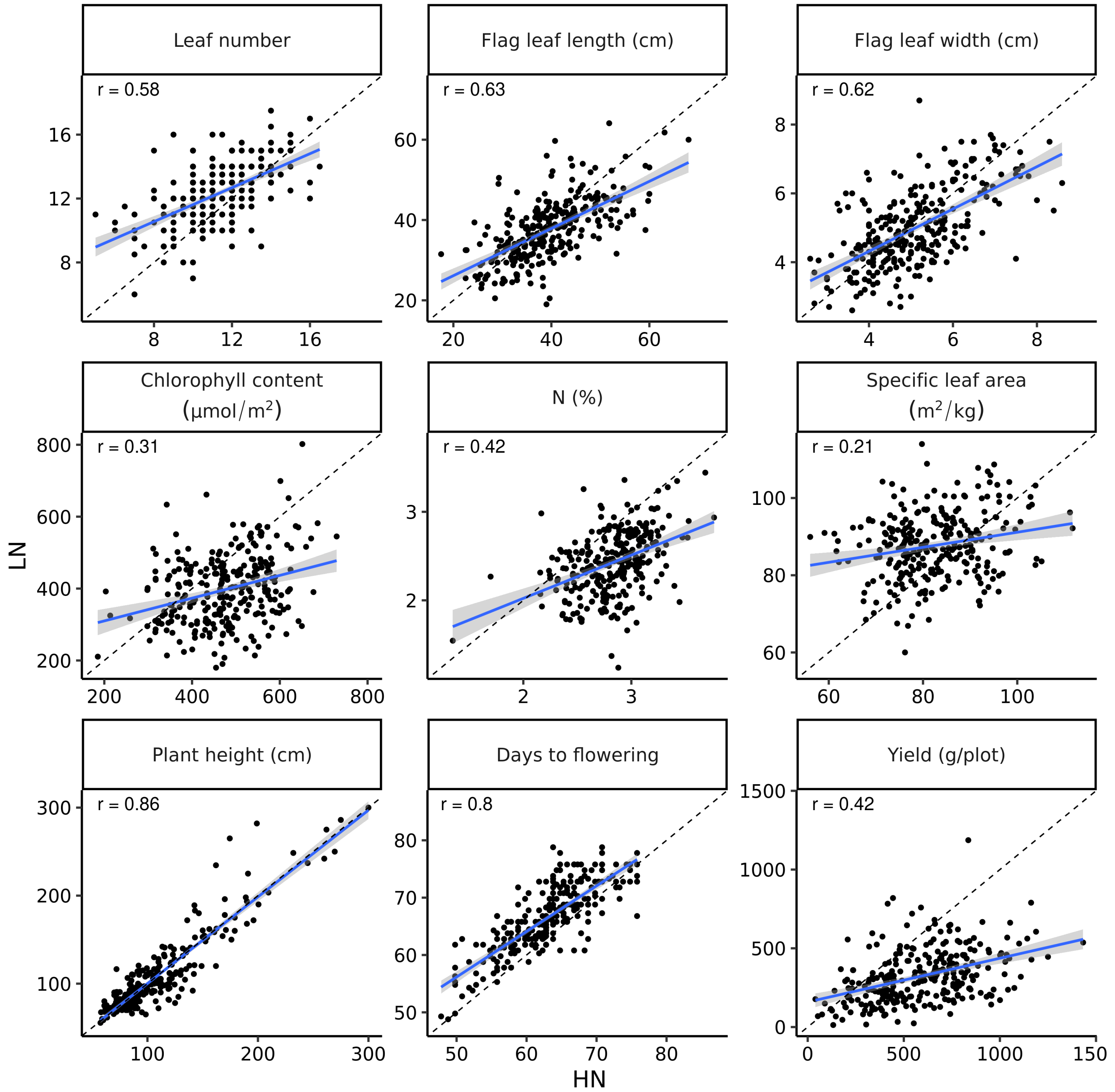


**Figure S3.** Scatter plot of genotype mean of morpho-physiological traits between two nitrogen conditions. r indicates Pearson correlation value. HN - high nitrogen, LN - low nitrogen.

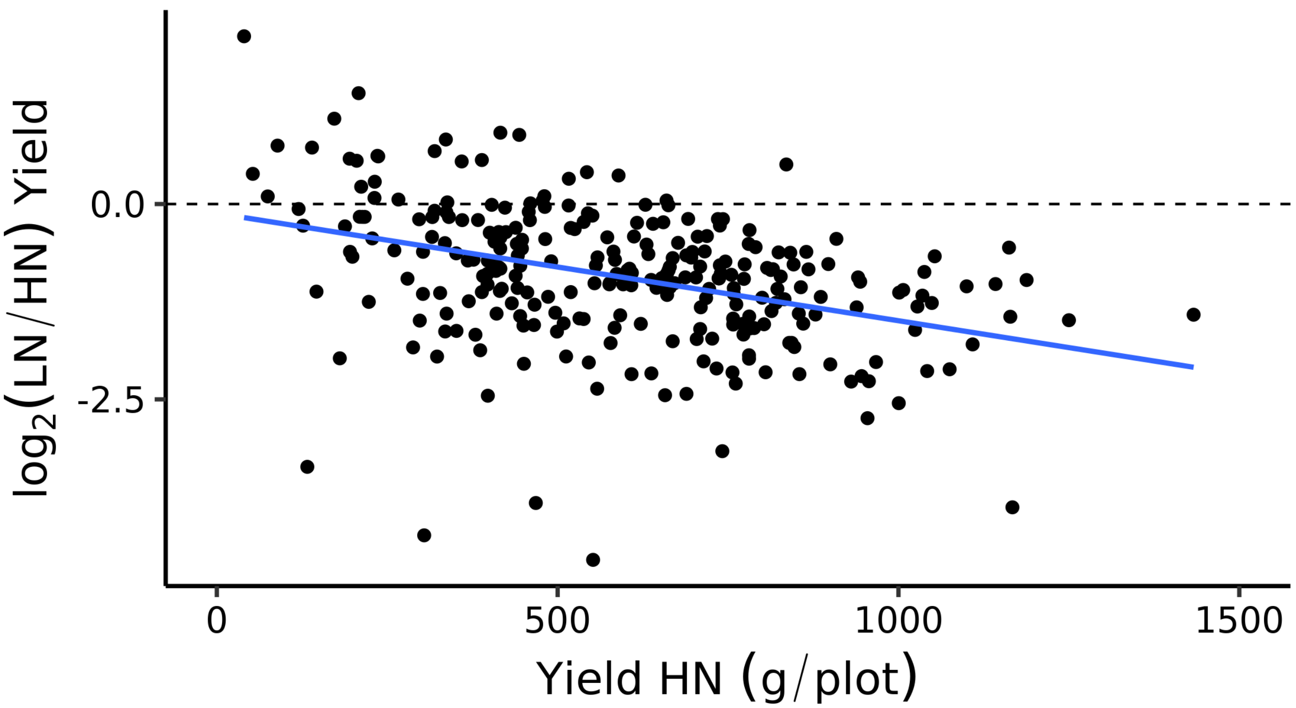


**Figure S4.** Plasticity in yield under low nitrogen (LN) stress versus yield at control (HN - high nitrogen) conditions. The dotted line indicates zero difference (no low nitrogen effect), while the solid blue line is the fitted regression. Each dot represents a genotype mean.

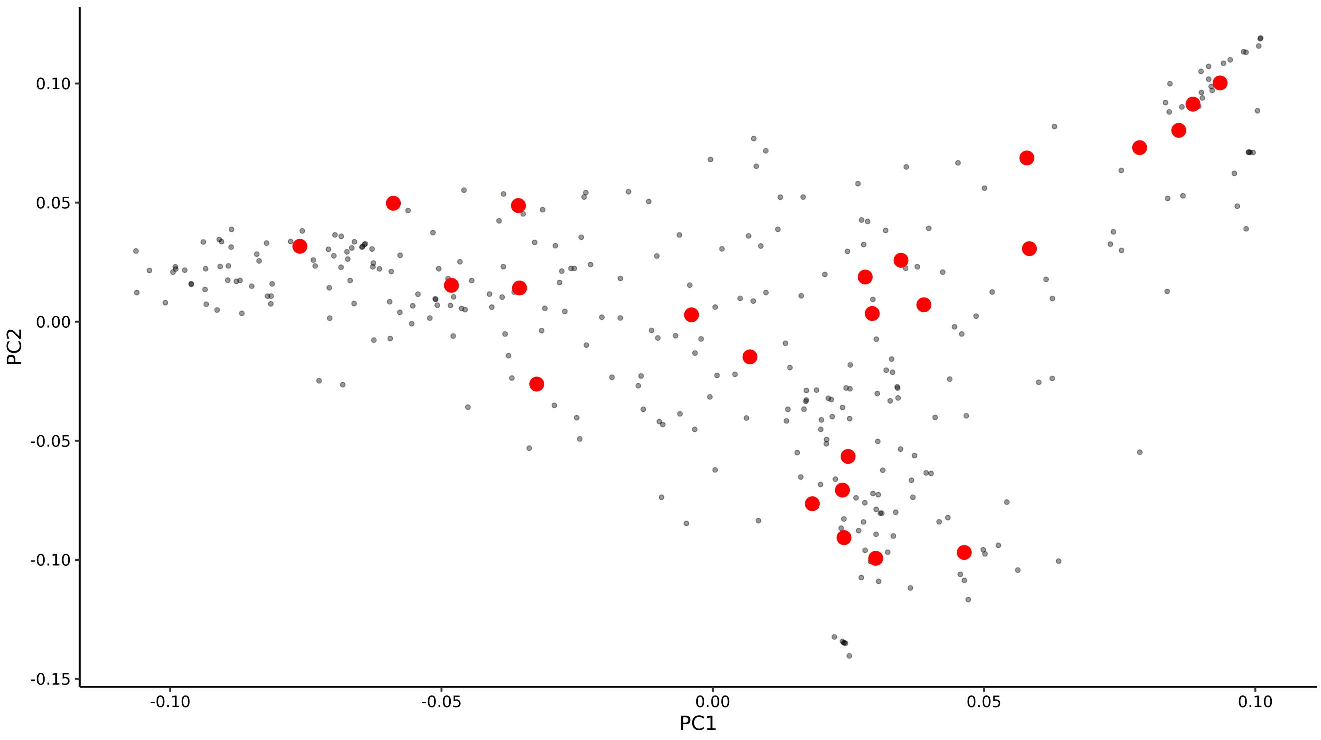


**Figure S5.** First two principle component from PCA based on SNPs from **?**. Each dot represent single genotype and red dots marked genotypes selected to metabolomic analysis.

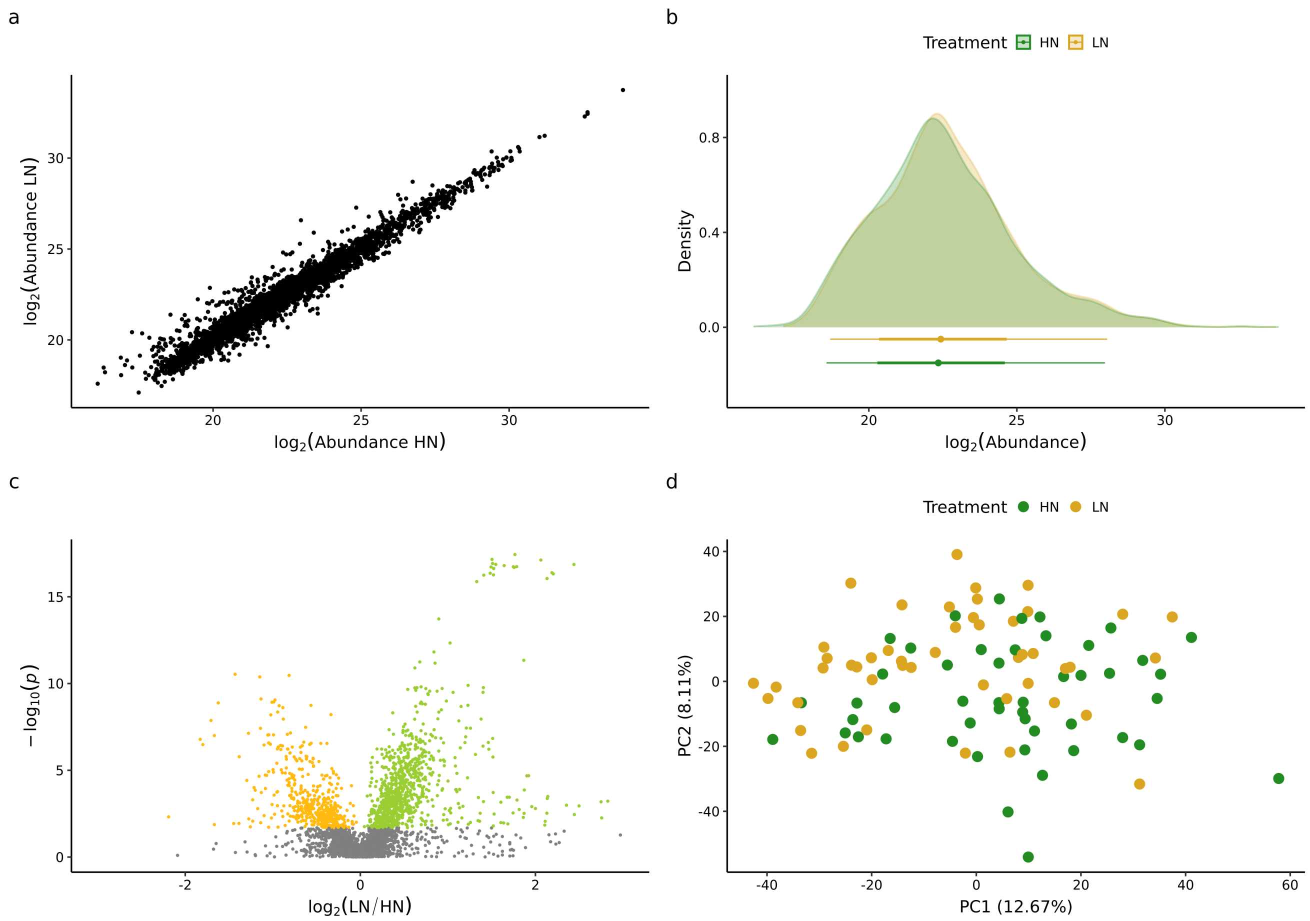


**Figure S6.** Metabolomics profiling in 24 sorghum genotypes across two nitrogen conditions based 3,496 identified compounds. **a** Scatter plot of abundance of 3,496 identified compounds across two treatment conditions. **b** Distribution of 3,496 identified compounds treatment conditions **c**. Volcano plot showing the downregulated (yellow) and upregulated (green) metabolites under low nitrogen (LN) conditions compare to high nitrogen (HN). **d** First two principle components (PC) from PCA based on 3,496 identified compounds. Values in bracket indicate amount of variance explained by each component. HN - high nitrogen, LN - low nitrogen.

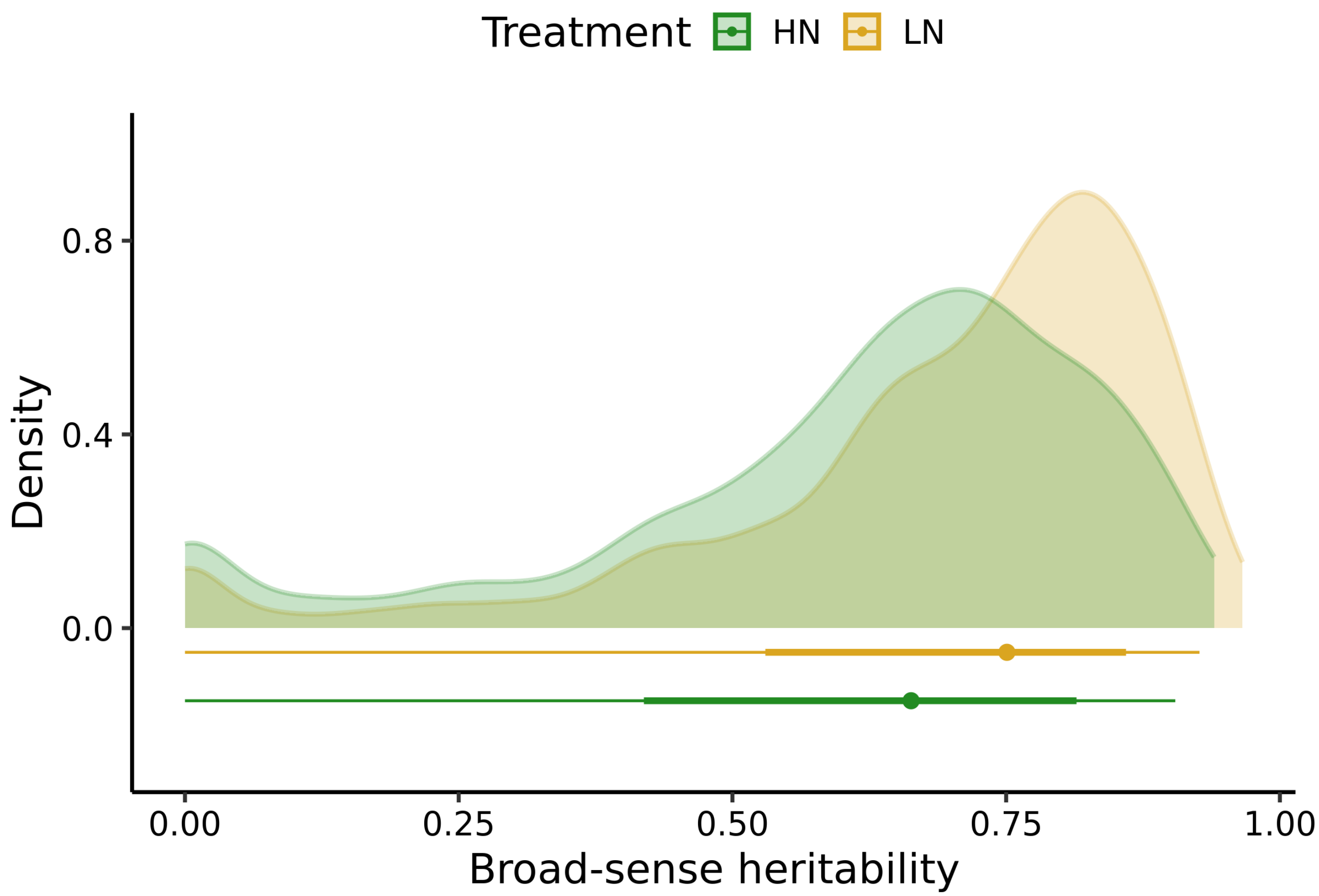


**Figure S7.** Distribution of broad-sense heritability ($H^2$) values for 3,496 identified compounds in two treatment condition. HN - high nitrogen, LN - low nitrogen.

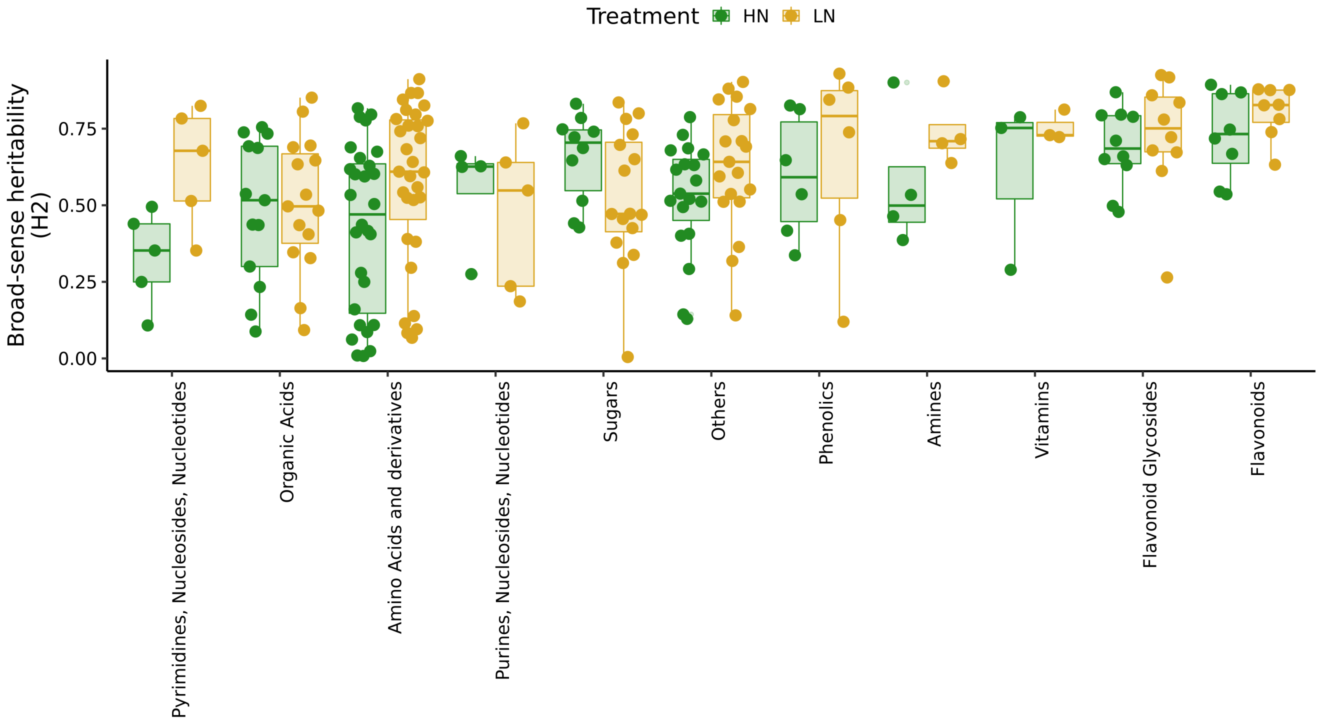


**Figure S8.** Broad-sense heritability ($H^2$) values for 145 annotated metabolites across 11 classes. Each dot indicate a single metabolite. HN - high nitrogen, LN - low nitrogen.

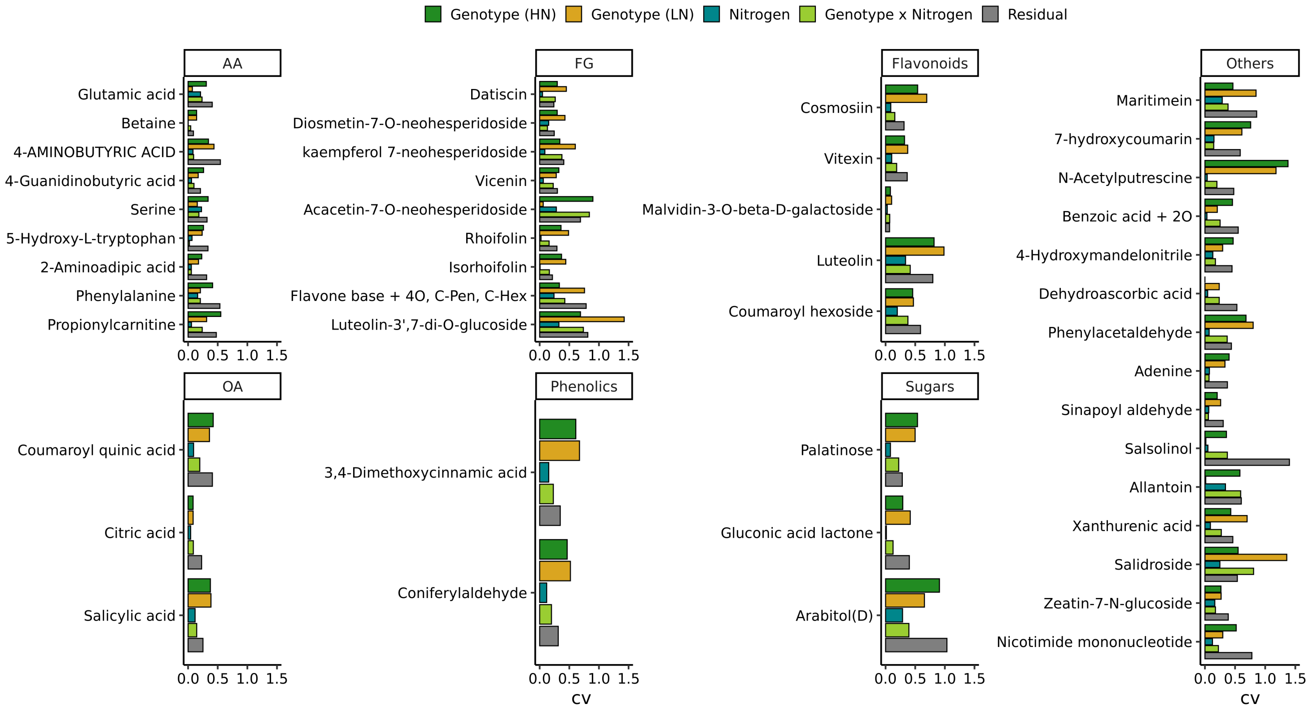


**Figure S9.** Coefficient of variation for 45 metabolites (CV; the estimated variance divided by the squared mean of the respective trait).

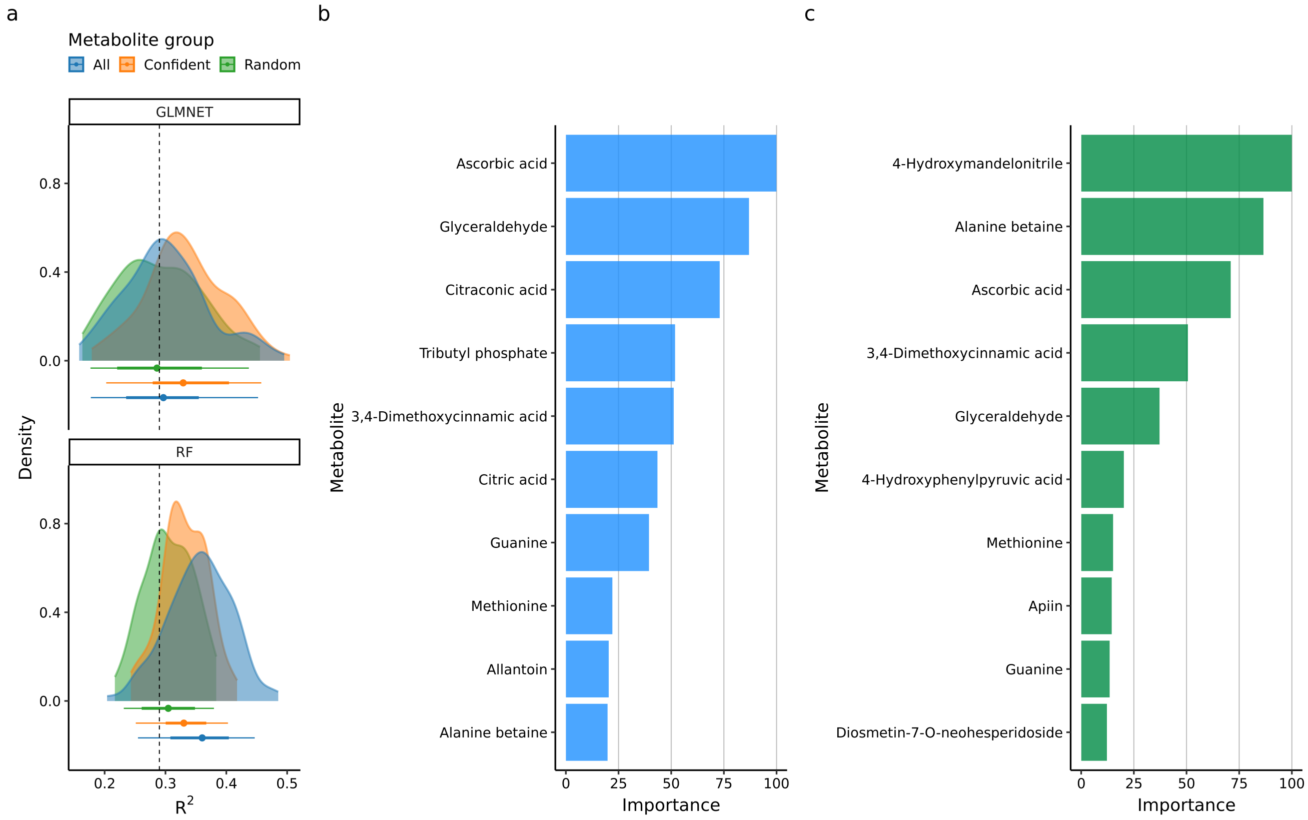


**Figure S10.** **a** Yield accuracy prediction based on three metabolites sets: all identified metabolites (n=3,496), metabolites with confident annotation (n=145) and the same number of metabolites with unknown annotation (n=145). $R^2$ were obtained from 100x repeated five-fold cross-validation. Dashed lines indicated $R^2$ values from regression based on treatment conditions. GLMNET - elastic-net regression, RF - random forest. Imporance values based on permutation for GLMNET(**b**) and RF(**c**).
